# Development of a web-based tool for undergraduate engagement in medical research; the ProjectPal experience

**DOI:** 10.1186/s12909-018-1272-5

**Published:** 2018-07-13

**Authors:** Timothy M. Rawson, Prasanthi Sivakumaran, Rhannon Lobo, Gheed Mahir, Adam Rossiter, Jeremy Levy, Alison H. McGregor, Martin Lupton, Graham Easton, Dipender Gill

**Affiliations:** 1 Imperial College London, South Kensington, London, SW7 2AZ UK; 2 Imperial College Healthcare NHS Trust, St Mary’s Hospital, London, W2 1NY UK; 3 Imperial College London, Hammersmith Hospital, Du Cane Road, London, W12 0NN UK

**Keywords:** Undergraduate medical research, Web-based interventions, Academic skills, Undergraduate engagement, Medical research

## Abstract

**Background:**

We report the development and evaluation of a web-based tool designed to facilitate student extra-curricular engagement in medical research through project matching students with academic supervisors.

UK based university students were surveyed to explore their perceptions of undergraduate research, barriers and facilitators to current engagement. Following this, an online web-based intervention (www.ProjectPal.org) was developed to support access of students to research projects and supervisors. A pilot intervention was undertaken across a London-based university in January 2013 to February 2016. In March 2016, anonymised data were extracted from the prospective data log for analysis of website engagement and usage. Supervisors were surveyed to evaluate the website and student outputs.

**Results:**

Fifty-one students responded to the electronic survey. Twenty-four (47%) reported frustration at a perceived lack of opportunities to carry out extra-curricular academic projects. Major barriers to engaging in undergraduate research reported were difficulties in identifying suitable supervisors (33/51; 65%) and time pressures (36/51; 71%) associated with this. Students reported being opportunistic in their engagement with undergraduate research. Following implementation of the website, 438 students signed up to ProjectPal and the website was accessed 1357 times. Access increased on a yearly basis. Overall, 70 projects were advertised by 35 supervisors. There were 86 applications made by students for these projects. By February 2016, the 70 projects had generated 5 peer-review publications with a further 7 manuscripts under peer-review, 14 national presentations, and 1 national prize.

**Conclusion:**

The use of an online platform to promote undergraduate engagement with extra-curricular research appears to facilitate extra-curricular engagement with research. Further work to understand the impact compared to normal opportunistic practices in enhancing student engagement is now underway.

**Electronic supplementary material:**

The online version of this article (10.1186/s12909-018-1272-5) contains supplementary material, which is available to authorized users.

## Background

In the United Kingdom (UK), the General Medical Council advocates that all trainees qualify from medical school with a grounded understanding of research regardless of whether they intend to pursue an academic career [1]. Knowledge of medical research is believed to promote trainee understanding of evidence-based medicine and facilitates critical analysis and interpretation of the medical literature that guides everyday practice [1]. This has been supported by the British Medical Association who have stated that research, teaching and training are a priority for securing the future of the National Health Service [2]. Whilst this area is beginning to be addressed within medical school curricula, this often tends to be primarily theoretical with limited active participation in research programmes [3, 4]. Where these are offered, such as through intercalated Bachelor of Science programmes, these are often highly competitive and remain limited in number in the majority of medical school programmes [5].

Evidence supports the value of undergraduate engagement in medical research [6–9]. Engaging in research promotes transferable skills, including the development of investigative approaches to medical problems, as well as promoting ongoing interest in academia following medical qualification [6, 10]. Furthermore, undergraduate students engaging in research are productive, often delivering national presentations and peer-reviewed publications through their involvement in research [6, 8, 10].

Despite the benefits of engagement in undergraduate research and the opportunities available to students, several major barriers to participation in the field commonly occur. These include time constraints, lack of expertise, limited support from supervisors, and funding difficulties [11–15]. These factors have a negative impact on undergraduate engagement, and are often reported as a major reason for students not pursuing research opportunities despite being aware of the benefits associated with them [15].

A further challenge is the current lack of formal pathways for identifying and documenting engagement with undergraduate research, particularly when it is extra-curricular. In the context of the UK, students currently rely on ad-hoc opportunities that present themselves, or “cold” emailing potential supervisors that they have identified on the internet to facilitate engagement in extra-curricular undergraduate research [10].

Given the current landscape of undergraduate engagement with research and the potential challenges that this poses, we report the development of a web-based platform to facilitate undergraduate engagement in extra-curricular research opportunities. The tool aims to facilitate student connections with appropriate academic supervisors and projects to formalise extra-curricular academic involvement and remove one of the major barriers to engagement with research reported within our cohort.

## Implementation

### Identifying challenges for undergraduate engagement in research

A questionnaire (Additional file 1) was developed based on a previously validated survey undertaken by Nikkar-Esfahani and colleagues, with permission from the authors [14]. This aimed to; (i) determine local student perceptions of undergraduate research opportunities and (ii) allow students to provide details of ways in which they would seek out involvement in undergraduate extra-curricular research. The survey was translated into an electronic platform and circulated to all medical students studying at two UK universities by advertisement through their medical school weekly e-mail newsletter. Participants were entered into a prize draw, where one student was picked at random to win a £25 cash prize. Results of the survey were tabulated and analysed quantitatively with the research team meeting to review the results of the analysis and deciding upon the development of the intervention, ProjectPal.

### Development of www.ProjectPal.org

Following analysis of reported barriers to engagement with undergraduate research, the authors agreed that development of a web-based platform that aimed to facilitate matching of students and supervisors to research projects. This was further supported through horizon scanning of current similar interventions that was facilitated by a London based technology transfer partner. This aimed to identify any similar interventions and map out gaps in their current development. An iterative design and development phase was then undertaken engaging a number of stakeholders including, 8 medical students, 5 research project supervisors, and 6 university tutors. The underlying functionality and graphical user interface were developed iteratively with feedback from these initial stakeholders in a semi-structured format to optimise function for the end-user [16]. This took the form of one-to-one semi-structured interviews that were then analysed by two independent researchers (TMR & DG) using line-by-line coding to generate core themes based on the categories that emerged from the data. This was iterative, using a mixed inductive – deductive technique [17]. Through this analysis, several key themes emerged and were addressed during the design and development of our intervention [18].

### ProjectPal launch

Following development and local testing, the ProjectPal website was officially implemented within a UK based university School of Medicine and the local hospital NHS Trust, which are all incorporated within an Academic Health Science Network. This pilot took place between January 2013 and February 2016. This was done with the support from academic staff and acquisition of an Academy of Medical Sciences INSPIRE scheme grant funded through the Wellcome Trust (on 18th January 2013) [19]. ProjectPal was not formally advertised as the authors wished to understand how this new intervention would disperse through the social community over time [20]. This method was selected as it allows the assessment of the success of an intervention based on the level at which it is adopted by the community in which it is deployed [21]. The hope was that this approach would provide insight into how, why, and at what rate the intervention was adopted and spread through the community [21]. Project data was collected prospectively via the website using an automatic data log that allowed auditing of the website’s functions.

### Auditing of data

In March 2016, anonymised data from January 2013 to February 2016 was extracted from the prospective data log for analysis. This included, (i) the type of projects advertised (classified as literature based, audit, laboratory, clinical, medical education, and unspecified); (ii) user characteristics; and (iii) website use information, such as sign up times / dates, log in rates, times of use, advertising rates, and application and acceptance rates. Data were tabulated and graphically analysed to describe the user habits of the tool as it was adopted over time. Initially, preliminary supervisor feedback was sought from supervisors who had advertised projects on the website consistently over the 3-year pilot period to assess the potential impact of the website on promoting engagement with undergraduate research. An electronic survey was emailed to all supervisors requesting feedback on the number of projects completed and students’ academic achievements during this timeframe. Furthermore, free text space was allowed for comments on the positives and negatives of the intervention from the supervisor’s perspective. This email was sent three times at two week intervals.

## Results

### Identifying challenges for undergraduate engagement in research

Fifty-one clinical medical students responded to the questionnaire. Additional file 1 provides the full raw results of the survey. There was a wide spread in what specialties students wanted to pursue following medical school with the majority wanting to pursue careers in medical subjects 14/51 (27%). Furthermore, 9/51 (18%) were unsure, 8/51 (17%) wished to pursue careers in surgery, and 5/51 (10%) wished to pursue academic careers. Despite this, 50/51 (98%) reported that they believed gaining experience in extra-curricular academic projects was beneficial to them with 46/51 (90%) having explored this option since joining medical school. Moreover, 40/51 (78%) had approached someone about taking part in research with consultants (13/51; 25%) and research fellows (17/51; 33%) being the most frequently approached potential supervisors. The majority of students reported a dual purpose to their engagement as they wished to undertake extra-curricular research to both boost their *curricula vitae* (CV) and because they enjoy engagement in academic pursuits (37/51; 73%).

**Fig. 1:**
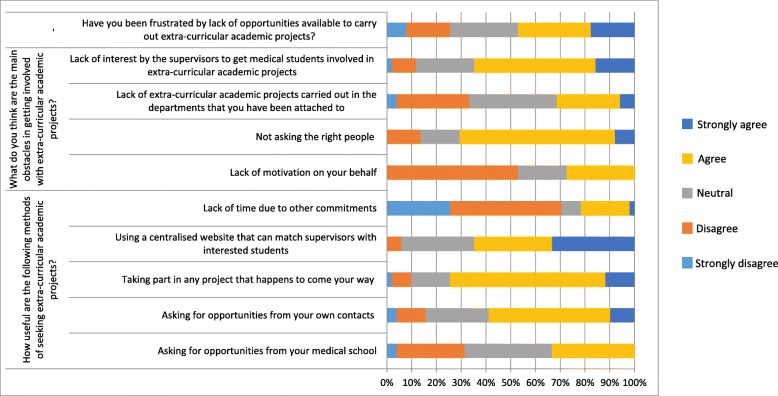
Summary of responses received from students regarding their agreement with statements on the current barriers and facilitators to engagement with extra-curricular academic research

Figure 1 summarises perceived barriers reported by respondents to engagement in extra-curricular research projects as undergraduate trainees. Twenty-four (47%) of respondents agreed or agreed strongly that they have been frustrated by a perceived lack of opportunities to carry out extra-curricular academic projects. Only 14/51 (27%) reported that a lack of motivation to pursue projects was a major obstacle to engagement. In contrast, 36/51 (71%) respondents agreed with that time pressures often were a major barrier and 33/51 (65%) agreed that supervisors often did not show interest in supporting students with their projects. On exploration of current methods that students use to seek out extra-curricular academic opportunities, 38/51 (75%) respondents reported that they tend take an opportunistic approach to engaging in undergraduate research. Furthermore, they agreed with statements that a central website to match them with appropriate supervisors (32/51, 63%) and seeking the ability to seek opportunities through contacts they have made (30/51, 59%) would be useful mechanisms for engaging more broadly with extra-curricular research.

### Development of ProjectPal

The current challenges that students reported regarding engagement in undergraduate research guided the development of ProjectPal. Firstly, development of a tool to facilitate undergraduate connections with supervisors appeared feasible, given the high level of students reporting an interest in engaging in undergraduate research. Secondly, the tool needed to be accessible to a wide range of students and supervisors to facilitate the broad interests in research and career pursuits that were reported. Thirdly, the intervention needed to ensure that students could connect with appropriate supervisors, but also act as a mechanism for formally recording their engagement in projects. This would support their needs for formal recognition for building evidence for their CV. Horizon scanning identified current solutions and potential gaps. Whilst there are a number of database based electronic tools to facilitate student-supervisor connections at academic institutes in North America, there was a major gap in translation of these systems outside of their host institutions [22, 23]. Furthermore, methods for linking formal engagement to outputs of research, such as electronic portfolios, was often not available for undergraduates. In cases where this type of resource was available, they tended to focus predominantly on post-graduate training.

**Fig. 2:**
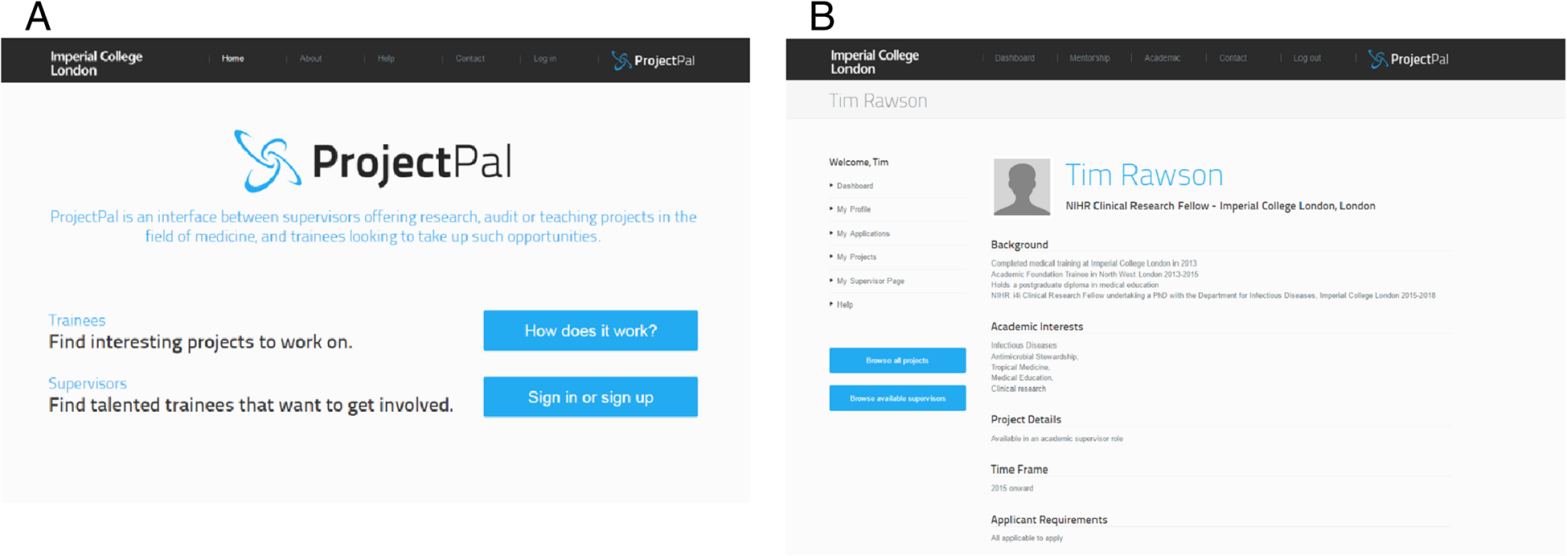
Key features of the ProjectPal website. **a** – Login page to ProjectPal; **b** example supervisor page with all navigation panels visible. All stakeholders agreed that ProjectPal must be designed to be straightforward to use. The site’s minimalist layout cuts down on superfluous text or controls, and was reported to make its use intuitive. There are a limited number of controls on each page which lends to a spacious design. This was reported to be easy on the eye and allowed the user to readily discern which feature they want to use. Although the layout is simple, the features were felt to be powerful. This comes from development of a streamlined flow of information, so that large amounts of detail are output in an easily accessible format. Applicants were able to browse and search through a simplified overview of projects, with the full details a click away. Similarly, supervisors positively reviewed the ability to quickly attain a summary of applications to their projects. Information about the status of applications is easily accessible by direct view on the ‘dashboard’, with e-mail notifications of any developments also offered following feedback from stakeholders. When creating profiles, website users are invited to offer their contact details, as well as some information on their current role, background and academic interests. When uploading projects, supervisors are required to provide details on the background, specific information on what the project will involve, as well as the time frame and applicant requirements. In turn, applicants applying for specific projects are required to offer a cover letter and a CV. Information and research governance was felt to be a significant issue surrounding the posting of projects by potential supervisors and senior management. Therefore, several rules must be adhered to on the webpage, with project information being anonymous and only available to NHS employees and medical students. Moreover, in line with the confidentiality and security requirements, projects may only be viewed or created by those with an authenticated log in, which in turn is only granted to those with an institutional e-mail address – either relating to employment within the National Health Service or affiliation with a UK medical school

Figure 2 demonstrates some of the features developed following horizon scanning and iterative stakeholder analysis. ProjectPal is a web-based application with a front-end of HyperText Markup Language (HTML), Cascading Style Sheets (CSS), and JavaScript interface. The backend of the device is a structured-query-language (SQL) database with Hypertext Pre-processor (PHP) server-side scripting used to communicate between the two. All data is stored in SQL databases with the PHP code used to store and retrieve data. The interface was designed to run across all internet browsers and is responsive to changes in screen size. This means that is adjusts according to whether it accessed from a computer, tablet or mobile phone. Navigation occurs through two navigation panels, one horizontal and one vertical. These were designed with stakeholder feedback to facilitate rapid movement between sections of the website.

The ProjectPal interface allows supervisors to post short descriptions of projects that they wish to advertise along with specific requirements (such as previous research experience or skills in statistical analysis) and anticipated outputs for students taking on the project (such as presentations or peer reviewed publication). Once the project is open for applications, users can browse for projects and can search based on supervisor/project location, keywords or experience required. Once a suitable or interesting project is found, the user may then submit an application. If they wish to apply, they are required to submit a short cover letter, statement of intent and their CV, which is then forwarded to the supervisor. The supervisor is notified of this via email and can login and choose to accept or reject the applicant. It is then for the supervisors to contact the applicant and decide whether or not the student is suited to the project in question.

### Website use

**Fig. 3:**
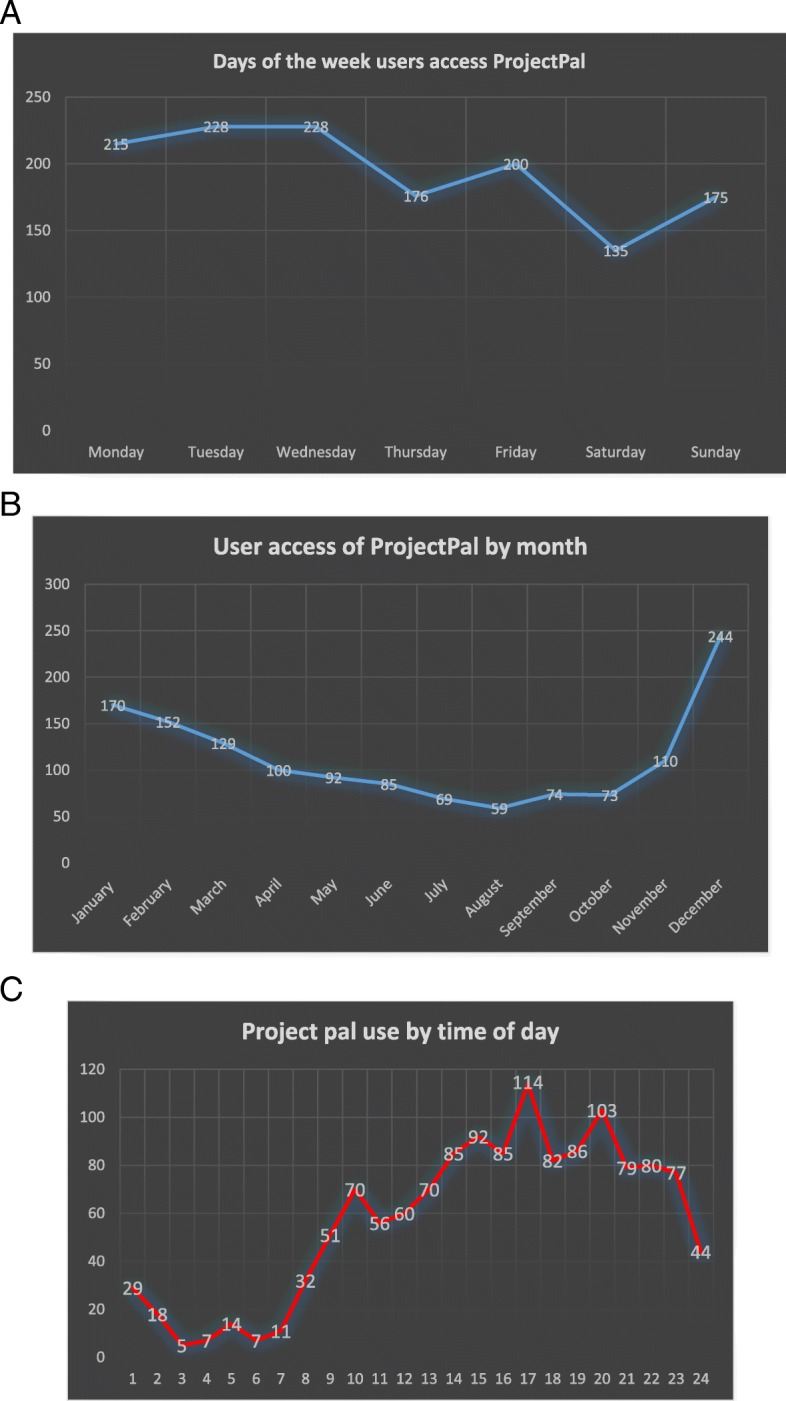
Trends in log in times. **a** ProjectPal website access on different days of the week between January 2013 and February 2016. **b** ProjectPal website access during different months between January 2013 and February 2016. **c** ProjectPal website access during different times of day between January 2013 and February 2016

Between January 2013 and February 2016, 438 individuals signed up to ProjectPal with the website accessed 1357 times in total. This is in a medical school with approximately 2000 undergraduate trainees. Figure 3 demonstrates trends in login times in terms of use by month, day and time of day, respectively. Access to the website increased on a yearly basis from 283 log-ins in 2013, 385 in 2014, and 488 by the end of 2015. In the 2 months included in the analysis for 2016 there had already been 201 log-ins to the website. The majority of access to the website tended to occur over winter, with December alone representing 244/1357 (18%) of all log-ins over the three full years analysed. Furthermore, the majority of log-ins tended to occur between Monday and Friday (1047/1357, 77%) and in the afternoons between 16:00 and 21:00 h.

### Project applications

Overall, 70 projects were advertised by 35 supervisors. However, 35 of the 70 (50%) projects were advertised by 4 supervisors. Two of these were involved in development of *ProjectPal* (DG & TMR). The most common type of projects advertised were literature review based (36/70, 51%). Table 1 outlines the characteristics of applicants and the projects that they applied for. In total, 86 individual applications were made for a wide variety of projects. The most popular projects applied for were in the specialties of neurology (17/86, 20%), infectious disease (17/86, 20%), and medical education (14/86, 16%), reflecting the specialist interests of supervisors who frequently engaged with the website. The majority of applications were from males (53/86, 62%) and the majority of applicants were in years 2 to 5 of undergraduate medical training (61/86, 71%), which runs for a total of 6 years for undergraduates at the university medical school. However, applications were also made by non-medical trainees (2/86, 2%) and a qualified medical doctor (1/86, 1%). The majority of applications (44/86, 51%) reported having no prior research experience before applying on ProjectPal.

**Table 1:** Website applicant characteristics

Characteristic		*n* = (%)
Project applications made	1st Year Medical Student	2 (2)
	2nd Year Medical Student	11 (13)
	3rd Year Medical Student	25 (29)
	4th Year Medical Student	14 (16)
	5th Year Medical Student	11 (13)
	Final Year Medical Student	4 (5)
	Graduate Entry Medical Student	6 (7)
	Health Care Professional	2 (2)
	Master of Research Student	1 (1)
	Medical Doctor	1 (1)
	PhD student	2 (2)
	Unspecified	7 (8)
Gender of applicants	Male	53 (62)
	Female	28 (33)
	Unknown	5 (6)
Previous research experience	Writing Scientific Abstracts	5 (6)
	Writing Literature Reviews	6 (7)
	Publication	6 (7)
	Previous Research Experience	19 (22)
	Poster Presentation	4 (5)
	None	44 (51)
	Lab Experience	9 (10)
	Experience of using reference manager software	3 (3)
	Clinical Audit	14 (16)
	Basic Understanding of Statistics	10 (12)
Specialty applied to	Endocrinology	3 (3)
	Immunology	6 (7)
	Infectious Diseases	17 (20)
	Maxillofacial surgery	1 (1)
	Medical Education	14 (16)
	Mental Health	1 (1)
	Musculoskeletal	5 (6)
	Neurology	17 (20)
	Oncology	2 (2)
	Other	1 (1)
	Pharmacology	4 (5)
	Primary Care	1 (1)
	Renal Medicine	1 (1)
	Respiratory	1 (1)
	Sports and Exercise Medicine	3 (3)
	Technology	2 (2)
	Unspecified	7 (8)

### Supervisor feedback

Supervisor engagement with requested feedback in this pilot evaluation of the website was poor after three rounds of emailing, with only three respondents. However, these were frequent users of the website representing 32/70 (46%) posted projects between January 2013 and February 2016. In total, supervisors reported that to date students that they had supervised had achieved; (i) 5 PubMed listed publications with 6 students named authors on these; (ii) 7 further manuscripts either undergoing peer-review or in press; (iii) 8 international academic presentations made by 10 students; (iv) 6 national presentations made by 7 students; and (v) 1 national prize of £500 awarded to 1 student. Furthermore, the supervisors reported that many of the students applying through ProjectPal were planning on to continue with further research projects following on from their initial applications.

## Discussion

We report the successful development of an online platform for the promotion of undergraduate engagement in research. Since January 2013, the website has grown in terms of users and the number of projects advertised as this concept diffuses through the networks that make up a large UK university and its associated Academic Health Science Centre. The majority of applicants for projects are currently medical students with a core number of supervisors tending to utilise the intervention to advertise projects at present.

The primary aim of this intervention was to better facilitate the connecting of students and supervisors to research projects of interest. This was focused upon to address the major barriers identified within this study survey and existing literature, such as the opportunistic nature of research projects, the time constraints associated with finding them, and difficulties in identifying appropriate supervision [11–15]. Whilst our results demonstrated a significant interest from students in engaging in undergraduate research for both academic and CV development, a major barrier identified was the lack of formal pathways for connecting with supervisors offering projects of interest. Furthermore, there was a need to formalise engagement with extra-curricular undergraduate research to provide better evidence of achievements outside of formal curriculum based activities. Whilst a number of database based connection tools were available in North America and electronic portfolios are made available for post-graduate students, there remained a paucity of tools to support undergraduates in the UK.

Following deployment of our intervention between 2013 and 2016, usage of the website tended to peak over the winter months, falling over student summer months. Furthermore, the website was predominantly accessed during the week between the hours of 16:00 and 21:00 h. This data suggests the aim of this resource was achieved, which was to support extra-curricular engagement with research opportunities. The majority of access happened outside “normal working hours” but also did not appear to impact on student’s weekends. The increase in activity during December may be explained by the students planning summer projects in advance of their holidays before they started exam revision. However, further longitudinal follow up is required to demonstrate the temporal relationship implied by this data.

By not advertising the website, we hoped to gain a basic understanding of uptake of the intervention and how use of it developed as it diffused throughout the social context in which it was deployed [17]. Within this study we observed that the intervention permeated successfully throughout undergraduates in the college. During this pilot roll out period, a diverse range of individuals applied through the ProjectPal website (www.projectpal.org) from encompassing pre-clinical medical students and postgraduates with increasing usage of the website on a yearly basis. Furthermore, although infectious diseases, neurology, and medical education were the specialties that were consistently applied for, there was a broadening range of projects from numerous clinical specialties advertised and applied for during deployment. Furthermore, students did not appear to apply for projects only in the specialty that they wished to pursue in the future [24]. This may have the added benefit of promoting a broader understanding of subjects that students may not be exposed to in detail at medical school [24].

Currently postgraduate researchers and clinicians have a wide variety of means of recording their academic achievements, through portfolio and e-portfolio systems [25]. These are often a requirement of their formal training pathways [25]. For undergraduate students and medical schools however, there are few formal mechanisms by which undergraduate research engagement and achievements can be recorded and assessed. Although the intervention was designed to facilitate undergraduate engagement with research, it has also provided a vehicle through which to capture and record the achievements of its students engaging in activities outside of the university’s curriculum. Current evidence supports the difficulties in quantifying successful outcomes from such undergraduate research projects as it is often seen to be labour intense and inefficient, with follow up not always occuring on completion of extra-curricular research [26]. The use of an online intervention with the ability to capture this data may offer a potential avenue to bridge this current gap and may help universities and organisations in supporting greater academic support and funding for their undergraduate members to engage in such activities in the future. This aspect of the website is now being further investigated as part of a larger longitudinal evaluation.

For the success of such an intervention it is also important to consider the potential outreach of such a tool beyond the local environment. The design of the system has been created to provide the optimal level of flexibility for use across different contexts and informatics systems. This was developed in this fashion as it was intended initially to be open access with data sharing across all users. However, there are also practical considerations to the dissemination of such tools, including cost of maintenance and control of sensitive information. This includes concerns over sharing of personal details and academic project ideas outside of the local university context. Following the successful pilot of this tool at our local university, we are now undertaking further work to explore the dissemination and implementation of the tool in different educational contexts, including a south-east Asian university medical school. This will provide evidence for the generalisability of ProjectPal as well as highlight further considerations for the development of open access tools to promote wider undergraduate engagement with research.

This study had several limitations. Further work is required to understand whether the students engaging with the website would have found projects of a similar level in its absence to better quantify the impact of the intervention for promoting undergraduate engagement in research. This is once again challenging to do given the paucity of pre-intervention data collection and a lack of systems available for undergraduate students to record their experiences prior to deployment of ProjectPal within this setting. This made comparison of the outcomes achieved through our intervention difficult as no formal mechanisms for recording undergraduate extra-curricular research output are currently available in this setting. Further prospective qualitative assessment of the tool may allow us to investigate student reasons for using the website and how this has changed the way that individuals approach engaging in undergraduate research projects.

Whilst the tool has been engaged with by a wide range of students, supervisor engagement has not matched this. Although 70 projects were advertised on the website during this time, 50% of these were posted by four individual supervisors. This along with poor levels of supervisor engagement with feedback from the pilot evaluation has made it challenging to understand current barriers and future facilitators to repeated project posting by supervisors on the website. We are now planning further in-depth analysis to understand the issues experienced by supervisors and how to improve supervisor acceptance of the tool.

Furthermore, by not advertising the intervention (for the reasons discussed above) we may have missed the opportunity to engage with a broader range of both students and supervisors.

## Conclusion

The use of an online platform to promote undergraduate engagement with extracurricular research has been implemented at a UK based university linked to an Academic Health Science Network with broad uptake from a diverse range of students. Furthermore, engagement through this website has generated a notable number of outputs for undergraduates engaging with the website. This includes several peer-reviewed publications, national/international presentations, and national prizes. Further work is now needed to better understand undergraduate and supervisor needs and incorporate these into the intervention for greater dissemination and uptake. The role of broadening the scope of such tools to facilitate researcher connections with other aspects of academia must also be explored. This includes post-graduate academic connections, such as for connecting peer-reviewers and skills sets within different levels of academic research.

## Availability and requirements

**Project name:** ProjectPal

**Project home page:** www.projectpal.org

**Operating system(s):** Platform independent

**Programming language:** Java

**Any restrictions to use by non-academics:** Permission needed from administrative staff

## Additional file

Additional file 1: Medical school questionnaire and responses. Full questionnaire and raw data responses received from survey of medical students during the development of ProjectPal. (DOC 131 kb)

## Abbreviations

CSS: Cascade style sheets
CV: Curriculum vitiae
HTML: HyperText Markup Language
PHP: Hypertext Pre-processor
SQL: Structured query language
UK: United Kingdom
