# Supplementary material for: Development of a web-based tool for undergraduate engagement in medical research; the ProjectPal experience
Source: BMC Med Educ. 2018 Jul 13;18:166. doi: 10.1186/s12909-018-1272-5 (PMC6044023; doi:10.1186/s12909-018-1272-5)
Supplement: Supplementary file 1 — Medical school questionnaire and responses. Full questionnaire and raw data responses received from survey of medical students during the development of ProjectPal. (DOC 131 kb) [file 12909_2018_1272_MOESM1_ESM.doc]

# Appendix 1 - Medical School Questionnaire and Responses

1) What specialty do you wish to pursue after graduation? (Choose only your top choice)

Medicine 14

Surgery 8

Paediatrics 4

Obs and Gynae 3

Psychiatry 1

Anaesthetics 3

Academic career 5

General Practice 2

Don’t know 9

Other 2 (management consultancy, ophthalmology)

2) Do you think experience with extra-curricular academic projects is useful for medical students?

Yes 50

No 1

3) Do you think experience with extra-curricular academic projects should influence selection for training jobs?

Yes 36

No 15

4) Have you been interested in carrying out extra-curricular academic projects since you started medical school?

Yes 46

No 5

5) Have you approached anyone about getting involved with extra-curricular academic projects?

Yes 40

No 11

6) Why would you want to carry out extra-curricular academic projects?

You enjoy research/audit/teaching 3

You want your CV to look better 7

Both 37

Other 4

Others:

Value of research (1)

Teach to consolidate my own learning (1)

Contributing to medical world (1)

Enjoy learning/teaching a new topic (1)

7) Have you been frustrated by lack of opportunities available to carry out extra-curricular academic projects? (‘1’ = strongly disagree, ‘5’ = strongly agree)

1. 4
2. 9
3. 14
4. 15
5. 9

8) What do you think are the main obstacles in getting involved with extra-curricular academic projects?

Lack of motivation on your behalf

1. 0
2. 14
3. 10
4. 27
5. 0

Not asking the right people

1. 4
2. 32
3. 8
4. 7
5. 0

Lack of extra-curricular academic projects carried out in the departments that you have been attached to

1. 3
2. 13
3. 18
4. 15
5. 2

Lack of interest by the supervisors to get medical students involved in extra-curricular academic projects

1. 8
2. 25
3. 12
4. 5
5. 1

9) How useful are the following methods of seeking extra-curricular academic projects?

Asking for opportunities from your medical school

1. 2
2. 14
3. 18
4. 17
5. 0

Asking for opportunities from your own contacts

1. 2
2. 6
3. 13
4. 25
5. 5

Taking part in any project that happens to come your way

1. 1
2. 4
3. 8
4. 32
5. 6

Using a centralised website that can match supervisors with interested students

1. 0
2. 3
3. 15
4. 16
5. 17

10) Would it be helpful if you were made aware of supervisors that were seeking medical students to help with extra-curricular academic projects?

Yes 51

No 0

11) Would you like to be notified of extra-curricular academic projects that you could participate in based on your preferences and level of commitment?

Yes 50

No 1

12) Would you be interested in using a website that could match you up with supervisors who need help with extra-curricular academic projects?

Yes 51

No 0

13) Have you been involved in any extra-curricular academic projects since the start of medical school?

Yes 39

No 12

14) How many extra-curricular academic projects have you been involved in since starting medical school?

| Of the 39 Clinical students who said yes:   | Number of projects | No. of students | | --- | --- | | 6 | 1 | | 3 | 6 | | 2 | 11 | | 1 | 18 | | 0 | 3 | |  |
| --- | --- | --- | --- | --- | --- | --- | --- | --- | --- | --- | --- | --- | --- |

15) What level in training was the individual who got you involved in extra-curricular academic projects?

| Of the 39 Clinical students who said yes:   | Level of training | Amount of students | | --- | --- | | SHO | 1 | | SpR | 6 | | Consultant | 13 | | Research Fellow | 17 | | Teaching Fellow | 2 | | PhD student | 2 | | Biotech Business | 1 | | Lab Scientist | 1 | | Medical student | 2 | | Other academics | 1 | | Non-medical scientist | 1 | | Lab technician | 1 | |  |
| --- | --- | --- | --- | --- | --- | --- | --- | --- | --- | --- | --- | --- | --- | --- | --- | --- | --- | --- | --- | --- | --- | --- | --- | --- | --- | --- | --- |

16) What specialty or specialties have you undertaken your extra-curricular academic projects in?

Of the 39 Clinical students who said yes:

| Specialty | No. of students |
| --- | --- |
| Neurology | 5 |
| Preclinical Med | 4 |
| Public health | 2 |
| Hpd surgery | 1 |
| Max Fax | 1 |
| Geratology | 1 |
| Orthopaedics | 2 |
| Respiratory | 1 |
| Physiology | 1 |
| FMRI | 1 |
| Sports science | 2 |
| Anatomy | 2 |
| Psychiatry | 2 |
| Radiology | 2 |
| Chemokines | 1 |
| Obs & Gynae | 2 |
| Colorectal | 2 |
| Reproduction | 1 |
| Urology | 1 |
| Neuropathology | 1 |
| Diabetes | 1 |
| Emergency medicine | 1 |
| Oncology | 2 |
| Genetics | 1 |
| Renal | 1 |
| Neonatal | 1 |
| General medicine | 1 |
| Surgery | 2 |
| HIV | 1 |
| Cardiology | 2 |
| Infectious disease | 1 |

17) Where did you carry out the extra-curricular academic projects?

Of the 39 Clinical students who said yes:

| Location | No. of students |
| --- | --- |
| FMRIB | 3 |
| College | 3 |
| Own computer/home | 2 |
| Oxford (unspecified) | 5 |
| John Radcliffe hospital | 6 |
| Biochemistry dept. | 1 |
| Medical school | 1 |
| Churchill | 2 |
| DPAG | 2 |
| NOC | 2 |
| Banbury (unspecified) | 1 |
| Radiology Dept. | 1 |
| Public (questionnaires) | 1 |
| Unspecified lab | 3 |
| London (unspecified) | 1 |
| Neuropathology dept. | 1 |
| Oxford Welcome Institute | 1 |
| Horton | 1 |
| GRAY lab | 1 |
| Library | 1 |
| Pathogen research building | 1 |

18) What part did you play in the extra-curricular academic projects?

Of the 39 Clinical students who said yes:

| Role | No. of students |
| --- | --- |
| Wrote up project | 8 |
| Taught | 5 |
| Data analysis | 11 |
| Data extraction | 1 |
| Investor | 1 |
| Clinical Trial | 1 |
| Auditing | 3 |
| Data collection | 9 |
| Case studies | 1 |
| Lab work | 3 |
| Experiments | 2 |
| Unspecified research | 3 |
| Databases | 1 |
| Screening titles for a review | 1 |

19) What has been the outcome of your extra-curricular academic projects?

Of the 39 Clinical students who said yes:

| Outcome | No. of students |
| --- | --- |
| Publication | 5 |
| Conference presentation | 1 |
| FHS dissertation | 4 |
| Posters | 6 |
| Presentations | 3 |
| Completed audit cycle | 2 |
| Paper | 2 |
| More info for supervisor | 2 |
| Prize | 1 |
| International conference | 1 |
| Not Finished | 8 |

20) If presented/published where were you on the list of authors?

Of the 17 presented/published

| Authorship | No. of students |
| --- | --- |
| Yes (unspecified) | 4 |
| 1^st^ author | 7 |
| 2^nd^ author | 2 |
| 3^rd^ author | 1 |
| 5^th^ author | 3 |
